# Supplementary material for: MetaRibo-Seq measures translation in microbiomes
Source: Nat Commun. 2020 Jun 29;11:3268. doi: 10.1038/s41467-020-17081-z (PMC7324362; doi:10.1038/s41467-020-17081-z)
Supplement: Supplementary file 10 — Supplementary Data 7 [file 41467_2020_17081_MOESM10_ESM.zip › File2/Confidence_High_Taxonomy/108424_out.krona.html]

Javascript must be enabled to view this page.

members
magnitude
magnitudeUnassigned
count
unassigned
taxon
rank

108424\_out

17

superkingdom
2
17

phylum
17
1239

class
909932
17

order
16
1843488

family
909930
16

genus
16
33024

species
16

SRS013158\_contig\_number\_contig-100\_7257.7257SRS013476\_contig\_number\_contig-100\_7967.140579SRS015960\_contig\_number\_6487SRS017859\_contig\_number\_contig-100\_24350.24351SRS019381\_contig\_number\_contig-100\_4413.45799SRS020394\_contig\_number\_8550SRS024492\_contig\_number\_contig-100\_3448.77779SRS024625\_contig\_number\_contig-100\_8139.66064SRS050520\_contig\_number\_contig-100\_20329.20330SRS050998\_contig\_number\_contig-100\_6878.6879SRS1041038\_contig\_number\_contig-100\_29417.65777SRS1041118\_contig\_number\_contig-100\_15548.54679SRS143085\_contig\_number\_contig-100\_14212.14213SRS145497\_contig\_number\_4136SRS149181\_contig\_number\_contig-100\_19471.109364SRS149879\_contig\_number\_16976
33025

1843489
1
order

1
31977
family

1
906
genus

species
1

SRS011302\_contig\_number\_22654
2292358
